# Root Canal Morphometry of the Maxillary and Mandibular Permanent Second Molars Among Mongolians: A Retrospective Study

**DOI:** 10.7759/cureus.93186

**Published:** 2025-09-25

**Authors:** Delgertsetseg Jargaltsogt, Urangua Erdenechuluun, Namuunzul Yondon, Enkh-Orchlon Batbayar, Oyuntugs Rashsuren, Batbayar Badral

**Affiliations:** 1 Department of Dental Hygiene, School of Dentistry, Mongolian National University of Medical Sciences (MNUMS), Ulaanbaatar, MNG; 2 Department of Oral and Maxillofacial Surgery, School of Dentistry, Mongolian National University of Medical Sciences (MNUMS), Ulaanbaatar, MNG; 3 Department of Oral and Maxillofacial Radiology, Central Dental Hospital, Mongolian National University of Medical Sciences (MNUMS), Ulaanbaatar, MNG

**Keywords:** c-shaped canal, melton’s classification, radiograph, root canal treatment, vertucci’s classification

## Abstract

Introduction

The overall success of root canal treatment depends on the dentist's knowledge of the anatomy of the root canal system, which is complex and varies in shape, making it difficult to remove organic tissue and reduce the microbial load - especially in the permanent second molar. A C-shaped canal configuration is most commonly found in the mandibular second molar. Therefore, careful radiographic evaluation may be helpful in diagnosing the root canal configuration prior to treatment. This study aims to examine the root canal morphometry of the maxillary and mandibular permanent second molars.

Methods

The retrospective study was conducted using CBCT images taken in the Department of Oral and Maxillofacial Radiology, Central Dental Hospital, Mongolian National University of Medical Sciences (MNUMS), Ulaanbaatar, Mongolia, after receiving ethical approval from the MNUMS Research Council (2024/3-02). CBCT images were selected according to the inclusion criteria and analyzed using OnDemand3D software (Cybermed Inc., Seoul, South Korea). The root canal morphometric parameters of the permanent second molar included the number of roots, the number of root canals, the root canal configuration based on Vertucci’s classification, and C-shaped canals based on Melton’s classification. The prevalence of C-shaped canal classifications was calculated across all selected cases. Statistical analysis was performed using IBM SPSS Statistics for Windows, Version 27 (Released 2019; IBM Corp., Armonk, NY, USA).

Results

Among the 96 CBCT images of individuals aged 15-57 who met the inclusion criteria for this study, a total of 332 permanent second molars - 174 maxillary and 158 mandibular - were analyzed. When we determined the number of roots and root canals of the permanent second molars, the findings revealed that 83 (25%) had a single-rooted tooth, 143 (43%) had two-rooted, and 105 (32%) had three-rooted teeth. Regarding the number of root canals, 21 (6.4%) of them had one, 103 (31.3%) had two, 191 (57.5%) had three, and 16 (4.8%) had four canals (p < 0.05). According to Vertucci’s classification, the root canal configuration of the maxillary second molars was as follows: Type I (84.2%), Type II (1.9%), Type III (0.2%), Type IV (11.3%), Type V (1.4%), and Type VIII (1%). The configuration for mandibular second molars included Type I (59.1%), Type II (4.6%), Type III (2.8%), Type IV (25.8%), Type V (5.4%), and Type VIII (2.3%). The prevalence of C-shaped canals in permanent second molars, classified using Melton’s system, was 11.4% in the maxilla and 29.1% in the mandible. According to the classification of C-shaped canal types, C1 type was found in 55%, C2 type in 18%, C3 type in 15%, and C4 type in 12% of cases.

Conclusion

Mongolians have mostly three-rooted teeth with three canals in the maxillary and two-rooted teeth with three canals in the mandibular second molars. Vertucci’s Type I canal configuration was predominant, and C-shaped canals were more frequently observed in the mandible than in the maxilla.

## Introduction

The overall success of root canal treatment depends on the clinician's knowledge of the anatomy of the root canal system, which is complex and varies in shape, making it difficult to remove organic tissue and reduce the microbial load [1]. In endodontic practice, clinicians should be aware of possible root canal anatomic variations, and the visualization of all canals is considered essential in endodontic therapy [2]. One of the most important anatomic variations is the “C” configuration of the canal system. The C-shaped canal, which was first documented in endodontic literature by Cooke and Cox in 1979, is so named for the cross-sectional morphology of the root and root canal [3]. The complexity of C-shaped canals makes them difficult to clean, shape, and obturate effectively [4]. A high prevalence of C-shaped root canals has been reported in mandibular second molars in the Asian population [5]. Currently, intraoral radiography is the imaging technique of choice for the management of endodontic disease, but CBCT imaging appears to have superior validity and reliability in the management of endodontic diagnosis and complications [6]. CBCT is a valuable tool for the diagnosis and treatment of root canal configuration in endodontics [7]. In Mongolia, the prevalence of dental caries and its complications is widespread, but the number of edentulous people is higher among the elderly than in the younger population [8]. Therefore, Mongolian dental clinicians need to save natural teeth through root canal treatments.

There have been insufficient studies evaluating the root canal morphometry of the permanent second molar teeth among Mongolian adults. This study aimed to use CBCT images to assess the root canal morphometry of the permanent second molar in Mongolian adults.

## Materials and methods

The study design was retrospective. Selected CBCT images were based on the following inclusion criteria: high-quality radiography, visible permanent second molar, age 15 years and over, clear visibility of the tooth’s anatomical structure, absence of any pathological signs and injuries, no presence of endodontic treatment, fully developed root, and Mongolian ethnicity. The exclusion criteria included the following: images that were blurred, those with secondary and false shadows, those of foreign citizens, those of immature teeth, and those indicating loss of hard tissue of the tooth or where the root was fractured. All images were taken in the Department of Oral and Maxillofacial Radiology, Central Dental Hospital, Mongolian National University of Medical Sciences (MNUMS), Ulaanbaatar, Mongolia, between 2021 and 2024. We used full CBCT (16 cm × 8 cm) images, which were obtained with HDX WILL (DENTRI, Seoul, Korea) (85 kW and 7 mA), using OnDemand3D (Cybermed Inc., Seoul, South Korea). The slice thickness on CBCT was 0.125 mm, and the rotation time was 17.5 seconds. A total of 332 permanent second molar teeth were examined using 96 CBCT images. To eliminate operator error, the two examiners performing CBCT measurements marked the cross-sectional plane; in 30 randomly selected cases, all measurements were made twice to assess intra-rater reliability across two-week intervals, using Cronbach's alpha (α = 0.98). The study was approved by the Research Ethics Committee of Mongolian National University of Medical Sciences (No. 2024/3-02).

**Figure 1:**
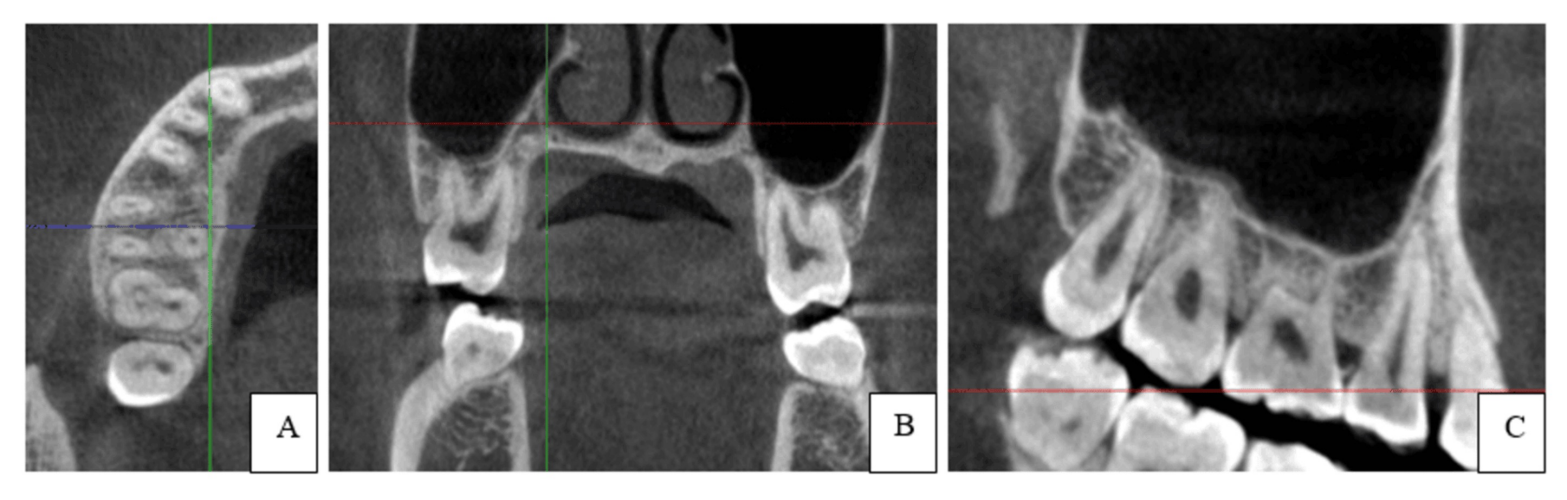
Maxillary Second Permanent Molar: Axial (A), Coronal (B), and Sagittal (C) Sectional Planes of CBCT Images Using OnDemand3D Software Image credit: Namuunzul Yondon

The number of roots and root canals of all second molar teeth were counted on the sagittal, coronal, and axial sectional planes of CBCT images, using OnDemand3D software (Figure 1). The root canal shape was classified according to Vertucci’s classification on the coronal and axial sectional planes, with Melton’s classification on the coronal sectional plane of CBCT images.

Statistical analysis

All data were evaluated using IBM SPSS Statistics for Windows, Version 27 (Released 2019; IBM Corp., Armonk, NY, USA). After checking for outliers in each measurement level (x ≤ Q1 - 1.5 IQR; x ≥ Q3 + 1.5 IQR), the Kolmogorov-Smirnov test was used to assess normal distribution. If the p-value was greater than 0.05, it was considered to follow a normal distribution. To calculate the differences in the quantity measurements of the number of roots and root canals of the second permanent molar teeth, Pearson’s Chi-square test was used. If the p-value was less than 0.05, the difference was considered statistically significant.

## Results

A total of 332 permanent second molar teeth were examined using 96 CBCT images of patients whose ages ranged from 15 to 57, and 63% of them were female. We analyzed 174 (52.4%) maxillary and 158 (47.6%) mandibular teeth.

Roots and canals of mandibular and maxillary second molars on CBCT sections

The results of the examination of the number of roots and root canals of the permanent second molar teeth, using CBCT images among Mongolian adults, are shown in Table 1.

**Table 1:** Comparison of the Number of Roots and Root Canals in Permanent Maxillary and Mandibular Second Molars *Pearson’s Chi-square test

		Number of root canals				Total number of roots	p-value*	χ²
Number of roots		1	2	3	4			
		n (%)	n (%)	n (%)	n (%)			
Maxilla	1	6 (3.4)	16 (9.2)	4 (2.3)	-	26 (14.9)	0.03	7.85
	2	-	13 (7.5)	29 (16.7)	-	42 (24.2)		
	3	-	-	92 (52.8)	13 (7.5)	105 (60.3)		
	4	-	-	-	1 (0.6)	1 (0.6)		
Total		6 (3.4)	29 (16.7)	125 (71.8)	14 (8.1)	174 (100)		
Mandible	1	15 (9.5)	35 (22.2)	7 (4.4)	-	57 (36.1)	0.02	6.40
	2	-	40 (25.3)	59 (37.4)	2 (1.2)	101 (63.9)		
	3	-	-	-	-	-		
	4	-	-	-	-	-		
Total		15 (9.5)	75 (47.5)	66 (41.8)	2 (1.2)	158 (100)		
Total number of root canals		21 (6.4)	103 (31.3)	191 (57.5)	16 (4.8)	332 (100)		

Three-rooted and three-root canal teeth were most commonly present in the maxilla (92, 52.8%), and four-rooted, four-root canal teeth were found in a single case, significantly. In the mandible, two-rooted teeth were most prevalent, and the presence of two- and three-root canal teeth was significantly higher.

Root canal shapes of permanent second molars by Vertucci’s classification on CBCT

We assessed the root canal shape of the maxillary permanent second molar according to Vertucci’s classification using CBCT images of Mongolian adults, as shown in Table 2.

**Table 2:** Root Canal Shapes of Maxillary Permanent Second Molars According to Vertucci’s Classification MB, Mesiobuccal; DB, Distobuccal; P, Palatal; B, Buccal

Maxillary permanent second molar						
Vertucci’s type	Three-rooted			Two-rooted		Single-rooted
	MB	DB	P	B	P	
	n (%)	n (%)	n (%)	n (%)	n (%)	n (%)
I	89 (84.8)	105 (100)	105 (100)	12 (28.6)	38 (90.5)	9 (34.6)
II	1 (0.9)	-	-	2 (4.8)	-	5 (19.2)
III	-	-	-	1 (2.4)	-	-
IV	13 (12.4)	-	-	25 (59.4)	4 (9.5)	6 (23.1)
V	2 (1.9)	-	-	2 (4.8)	-	2 (7.7)
VIII	-	-	-	-	-	4 (15.4)
Total	105 (100)	105 (100)	105 (100)	42 (100)	42 (100)	26 (100)

All maxillary permanent second molars were classified by the number of roots: three-rooted, 60.7% (105); two-rooted, 24.3% (42); and single-rooted, 15.0% (26). Vertucci’s type was predominantly Type I, but there were rare cases of Type III and Type V, which are difficult to treat with root canal therapy.

We determined the canal type of two-rooted mandibular permanent second molars: Type IV - 41 (40.7%), Type I - 38 (37.6%), Type II - 10 (9.9%), Type III - 7 (6.9%), and Type V - 5 (4.9%) in the mesial canals; and Type I - 95 (94%), Type IV - 3 (3.6%), Type II - 2 (1.7%), and Type V - 1 (0.7%) in the distal canals. However, in single-rooted permanent second molars, the most common types were: Type IV - 23 (40.4%), Type I - 20 (35.1%), Type V - 8 (14%), and Type VIII - 6 (10.5%) (Table 3).

**Table 3:** Root Canal Shapes of Mandibular Permanent Second Molars According to Vertucci’s Classification M, Medial; D, Distal

Mandibular permanent second molar			
Vertucci’s type	Two-rooted		Single-rooted
	М	D	
	n (%)	n (%)	n (%)
I	38 (37.6)	95 (94)	20 (35.1)
II	10 (9.9)	2 (1.7)	-
III	7 (6.9)	-	-
IV	41 (40.7)	3 (3.6)	23 (40.4)
V	5 (4.9)	1 (0.7)	8 (14)
VIII	-	-	6 (10.5)
	101 (100)	101 (100)	57 (100)

The examined permanent mandibular second molars were two-rooted (63.9%, 101) and single-rooted (36.1%, 57). The most common type of root canal was Vertucci’s Type I.

Assessment of permanent second molar root canal shape by Melton’s classification on coronal CBCT images

The prevalence of the C-shaped canal among all examined second molars was 19.9% (CI 95%, SD 15.6%-24.2%), with 11.4% in the maxillary and 29.1% in the mandibular molars. The distribution of C-shaped canal types according to Melton’s classification is shown in Table 4.

**Table 4:** Distribution of C-Shaped Canal Type by Melton’s Classification

Melton’s type	C1	C2	C3	С4	Total
	n (%)	n (%)	n (%)	n (%)	n (%)
Maxillary	10 (5.7)	2 (1.1)	2 (1.1)	6 (3.5)	20 (11.4)
Mandibular	26 (16.4)	10 (6.3)	8 (5.1)	2 (1.3)	46 (29.1)

## Discussion

This study aimed to evaluate the morphometry of the root canal of the permanent second molars using CBCT images and to provide clinicians with valuable information for endodontic treatment.

The most common root configuration in our study was three-rooted in the maxilla. The incidence of three-rooted maxillary second molars was 60.7%, whereas two-rooted molars accounted for 24.3%, and single-rooted molars for 15.0%. Four-rooted configurations were not observed in our study population, whereas the Saudi population exhibited a different distribution: 92% were three-rooted, 6.6% were two-rooted, 1.1% were four-rooted, and 0.3% were single-rooted [9]. In the Chinese population, 78.0% had three roots, and fused roots were observed in 31.5% of teeth [10]. A three-rooted maxillary second molar was found in 87.1% of the Thai population [11]. The most common configuration of mandibular second molars in our study was two-rooted (63.9%), which was similar to findings in the Indian population (79.5%) [12] and the Egyptian subpopulation (83.4%) [13]. 

In our study - morphologically speaking - of the root canals of the permanent second molar by Vertucci’s classification, 84.8% of mesiobuccal canals and 100% of distal and palatal canals in three-rooted maxillary second molars had Type I. In China, 7 (74.25%), 29 (7.25%), 9 (2.25%), and 65 (16.25%) teeth had Type I, II, III, and IV mesiobuccal root canals, respectively, with 103 (25.75%) having secondary mesiobuccal canals [10]. In India, the predominant canal morphology in the mesial roots was Vertucci's Type IV (45.17%), followed by Type II (32.55%), Type I (7.23%), Type V (1.02%), and Type III (0.91%). In contrast, the distal root showed Type I (61.14%) as the predominant canal configuration, followed by Type II (18.21%) and Type IV (7.53%) [12]. A Type I canal configuration (>75%) was the most frequent in all the roots of second molars in the Ugandan population [14].

The findings of a systematic review revealed a wide variety of possible canal configurations among permanent mandibular second molars, with the number of canals ranging from one to five. In the mesial root, the Type IV canal configuration was more frequent (37.3%), with two canals present in 70.4% of cases. In the distal roots, one canal was more prevalent (77%), and the Type I canal configuration was more frequent, at 85.2% [15]. In the Iranian population, the most prevalent canal configuration in the mesial root was Type II, while the distal root most commonly had Type I, according to Vertucci’s classification [16].

The prevalence of C-shaped anatomy in second molars depends on gender, geographic region, age, and tooth group. A meta-analysis revealed that no significant difference in the prevalence of C-shaped configurations was observed between sexes in mandibular second molars. The pooled proportion of C-shaped anatomy in mandibular second molars in East Asian countries (39.6%; 36.0%-43.1%) was significantly higher compared to other regions [17]. For example, in the Indian population, the prevalence of teeth with C-shaped root canals was 13.12% [12]; in Argentina, 11% [18]; in Nepal, 12.6% [19]; and in our study, the prevalence was 19.9%-11.4% in the maxillary and 29.1% in the mandibular molars.

To our knowledge, this is the first study concerning the morphology of second molar teeth in Mongolia. The main limitation of the study was the sample size, which may not be representative of the age groups, genders, and regional distribution in Mongolia. Future studies could be conducted with a larger sample size and more balanced age and gender groups. The study timeline may have limited the ability to track longitudinal effects. General dentists should refer patients who require root canal treatment to endodontists and recommend a CBCT examination.

## Conclusions

The root number of the permanent second molars in Mongolians showed significantly different results: most exhibited second molars with three roots in the maxilla and two roots in the mandible. The root canal configuration was predominantly Vertucci Type I, while Melton’s C1 type and C-shaped canals were more frequently observed in the mandibular permanent second molars than in the maxillary ones.
